# The pathogenesis of coronavirus-19 disease

**DOI:** 10.1186/s12929-022-00872-5

**Published:** 2022-10-26

**Authors:** Alain C. Borczuk, Rhonda K. Yantiss

**Affiliations:** 1 Department of Pathology, Donald and Barbara Zucker School of Medicine at Hofstra/Northwell, Greenvale, NY USA; 2 Department of Pathology and Laboratory Medicine, Weill Cornell Medicine, 525 East 68th Street, New York, NY 10065 USA

**Keywords:** COVID-19, SARS-CoV-2, Pathology, Histology, Mechanisms

## Abstract

Severe acute respiratory syndrome-associated coronavirus-2 (SARS-CoV-2) is the causal agent of coronavirus disease-2019 (COVID-19), a systemic illness characterized by variably severe pulmonary symptoms, cardiac conduction abnormalities, diarrhea, and gastrointestinal bleeding, as well as neurologic deficits, renal insufficiency, myalgias, endocrine abnormalities, and other perturbations that reflect widespread microvascular injury and a pro-inflammatory state. The mechanisms underlying the various manifestations of viral infection are incompletely understood but most data suggest that severe COVID-19 results from virus-driven perturbations in the immune system and resultant tissue injury. Aberrant interferon-related responses lead to alterations in cytokine elaboration that deplete resident immune cells while simultaneously recruiting hyperactive macrophages and functionally altered neutrophils, thereby tipping the balance from adaptive immunity to innate immunity. Disproportionate activation of these macrophages and neutrophils further depletes normal activity of B-cells, T-cells, and natural killer (NK) cells. In addition, this pro-inflammatory state stimulates uncontrolled complement activation and development of neutrophil extracellular traps (NETS), both of which promote the coagulation cascade and induce a state of “thrombo-inflammation”. These perturbations have similar manifestations in multiple organ systems, which frequently show pathologic findings related to microvascular injury and thrombosis of large and small vessels. However, the pulmonary findings in patients with severe COVID-19 are generally more pronounced than those of other organs. Not only do they feature inflammatory thromboses and endothelial injury, but much of the parenchymal damage stems from failed maturation of alveolar pneumocytes, interactions between type 2 pneumocytes and non-resident macrophages, and a greater degree of NET formation. The purpose of this review is to discuss the pathogenesis underlying organ damage that can occur in patients with SARS-CoV-2 infection. Understanding these mechanisms of injury is important to development of future therapies for patients with COVID-19, many of which will likely target specific components of the immune system, particularly NET induction, pro-inflammatory cytokines, and subpopulations of immune cells.

## Background

A novel human pathogen emerged when a seafood market worker in the Hubei province of China presented with fever, dizziness, and cough in December of 2020. One month later, there were 9976 similar cases reported across 21 countries and within a few more weeks, the infection had spread to every corner of the globe. The disease has since claimed well over six million lives worldwide and caused substantial morbidity among patients who survived their illnesses. Early in the pandemic, teams of investigators identified the causal agent of coronavirus disease-2019 (COVID-19) to be severe acute respiratory syndrome-associated coronavirus-2 (SARS-CoV-2). They subsequently demonstrated its relationships to two other beta-coronaviruses known to cause severe pneumonia in humans, SARS-CoV and MERS-CoV, as well as a high-degree of homology with a coronavirus found in bats [1].

The pathologic features of COVID-19 are variable in distribution and severity. Although most patients have upper respiratory tract and pulmonary symptoms, those with severe disease may also develop widespread small and large vessel thrombosis, microvascular injury, cardiac conduction abnormalities, neurologic deficits, diarrheal symptoms, and gastrointestinal bleeding, any of which can be life-threatening. The mechanisms underlying disease manifestations are incompletely understood, yet most data suggest that severe COVID-19 results from a combination of immune dysregulation and depletion of key immune cell subsets, complement activation, and interactions between the innate immune system and the coagulation cascade that promote inflammatory thromboses. The purpose of this review is to discuss the pathogenesis underlying organ damage that can occur in patients with SARS-CoV-2 infection.

## Viral structure and cell entry

SARS-CoV-2 is a positive-sense single-stranded RNA virus that contains four major types of proteins: nucleocapsid, membrane, envelope, and spike proteins. The spike protein is composed of two subunits, S1 and S2, that are required for viral entry into cells. The S1 subunit binds the angiotensin converting enzyme-2 (ACE2) receptor, whereas S2 is cleaved by transmembrane serine protease-2 (TMPRSS2), thereby facilitating viral fusion with the cell membrane [2]. Heavy glycosylation of spike protein shields it from the immune system and modulates the conformational dynamics of the receptor binding domain located on S1, thereby ensuring viral interactions with ACE2 [3]. The viral burden is highest in tissues expressing both ACE2 and TMPRSS2, such as epithelia of the tracheobronchial tree, type 2 pneumocytes (AT2 cells), endothelial cells, cardiomyocytes, and epithelial cells of the small bowel and colon. This distribution of cell susceptibility partly explains the constellation of symptoms commonly encountered among patients with COVID-19 [4–6].

In addition to ACE2 and TMPRSS2, other proteins can facilitate viral entry in a site-specific fashion. ACE2 is expressed in combination with cathepsin L in alveolar type 1 pneumocytes (AT1 cells) and AT2 cells, enterocytes, cardiomyocytes, and the placenta [7]. Cathepsins activate IL-1β and NLRP3-mediated inflammatory cascades that cause cell apoptosis or pyroptosis, and they play important roles in matrix remodeling, neutrophil recruitment, and emergency myelopoiesis. Viral attachment to the cell is facilitated by heparan sulfate, while furin-like cleavage sites on spike protein promote viral replication in the lung [8, 9]. Neuropilin1 (NRP1) binds these furin-derived substrates and assists viral entry into nasal cells [8, 9]. ACE2 can be expressed in combination with pro-inflammatory proteases (e.g., furin, PCSK5, and PCSK7) that tend to be expressed at higher levels in males and increase with age [10].

## Mechanisms of tissue injury due to SARS-CoV-2

Many of the clinical manifestations of SARS-CoV-2 infection are related to virus-driven perturbations in the immune system and resultant tissue injury. Combinations of these changes can be distilled into three distinct groups based on aberrant interferon-related responses, cytokine alterations, recruitment of functionally altered immune cells, and uncontrolled complement activation with associated neutrophil extracellular traps (NETS) and systemic thrombosis. These groups include (1) humoral immunodeficiency with B-cell defects, (2) a hyperinflammatory state characterized by loss of T-cell subsets and high cytokine levels driven by IL-6, IL-1β, TNF-α, and (3) complement-driven injury [11]. These mechanisms are described further below.

### Interferon-related responses

Interferon (IFN)-related responses represent important components of the host defense against viral infections. Interferons are broadly classified as types I, II, and III: type I IFNs bind to the IFN-α/β receptor, type II IFN (IFN-γ) is activated by IL-12, and type III IFNs signal through either IL-10R2 (IL-10 receptor 2 subunit) or IFNLR-1 (interferon lambda receptor 1) complexes. Although IFNs are important components of the fight against viral infections, they can also promote tissue injury when present in excess, indicating that timing and organ location determine the net effects of IFN-mediated immune responses [12]. Peripheral blood mononuclear cells show increased type I IFN profiles that correlate with viral loads early in the disease course, followed by decreasing type I IFN as viral levels fall. However, IFN-related responses tend to be reduced or delayed in patients with severe COVID-19 [13]. Suboptimal IFN-related responses in this setting may be due to genetic influences, neutralizing autoantibodies, or depleted numbers of plasmacytoid dendritic cells that produce TLR7, a toll-like receptor that detects single-stranded viral ribonucleic acids and promotes IFN-related responses [14].

Interferon levels vary among different tissues and tend to correlate with disease severity among COVID-19 patients. Type I IFN-related responses are important in early infection, although they are also persistently elevated among patients with sustained viral replication. Increased type II IFN is associated with severe disease at all sites, whereas type III IFN is mostly seen in the upper airways of patients with mild disease and high viral loads [15, 16]. It is possible that type III IFN-related responses in the upper airways are responsible for clearing SARS-CoV-2 but when anti-viral efforts fail, the balance shifts to a pro-inflammatory state sustained by types I and II IFN in the lower airways [17].

Data from animal models support the idea that severe COVID-19 is a consequence of failed IFN-driven anti-viral activities. Ferrets with pneumonia due to SARS-COV-2 usually clear the virus, rather than succumb to it. These animals have increased M1 macrophages with anti-viral profiles (e.g., IL-1β, CXCL8, and type I IFN) early in disease then switch to an anti-inflammatory M2 macrophage response in the resolution phases of disease [18]. On the other hand, aged ferrets have higher viral loads in their respiratory tracts, and they tend to develop more severe disease characterized by persistent T-cell activation and a M1 macrophage response without a timely switch to the M2 macrophage-mediated resolution phase. Persistent CXCL10 secreted by these macrophages functions as a chemoattractant for monocyte/macrophages, NK cells, and T-cells, thereby driving the inflammatory state [19]. These observations suggest that severe COVID-19 in humans may result from a persistent M1 response without the M2 shift.

### Cytokine responses

Compared with influenza, the pneumonia associated with SARS-CoV-2 infection features a greater degree of neutrophil degranulation and increased cytokine levels, particularly IL-1β, IL-1RA, TNF-α, G-CSF, CCL7, CXCL1, CXCL8, CXCL11, and CXCL12a [20]. SARS-CoV-2 infection can also precipitate macrophage activation syndrome, which is characterized by increased type II IFN-related responses as well as elevated IL-6, IL-1β, TNF-α, and ferritin levels [21]. Patients with severe COVID-19 typically have increased serum levels of IL-6, IL-8/CXCL8, CXCL9, CXCL10, TNF-α, MCP1/CCL2, RANTES/CCL5, IL-18, and MIP-1α/CCL3, which promote a sustained pro-inflammatory state that may persist for up to 60 days [22, 23].

### Cellular recruitment and functional perturbations

#### Innate immunity

SARS-CoV-2 infection promotes an imbalance between innate and adaptive immunity that becomes more pronounced when patients develop severe symptoms. Mild disease is characterized by an adaptive immune response with robust recruitment of plasmablasts and CD8 + T-cells [13]. However, more severe disease features a predominance of innate immunity with elaboration of inflammatory cytokines (e.g., IL-6, IL-1β and TNF-α) accompanied by lymphopenia and a decrease in CD4+ and CD8+ T-cells, as well as markers of T-cell exhaustion [14]. Patients with severe COVID-19 also have increased numbers of low-density neutrophils, which comprise a subset of dysfunctional granulocytes that is increased in autoimmune conditions and other inflammatory disorders [24]. Low-density neutrophils promote tissue injury by contributing to immune suppression, inducing thrombosis, and facilitating NET formation, as described below [25, 26].

Epithelial cells also elaborate MCP1/CCL2, CCL3, CXCL1, CXCL3, CXCL10, IL-8/CXCL8, IL-1β, and TNF-α; these cytokines recruit macrophages and further promote tissue damage [27]. As the disease progresses, normal alveolar macrophages are ultimately depleted and replaced by pro-inflammatory macrophages [28]. These HLA-DR-low/S100A-high monocytes are spatially related to epithelial cells, creating a sustained interface between epithelial and immune cells that promotes immune activation [12, 27, 29]. Similar alterations are seen in the lungs of African green monkeys, in which case viral replication in pneumocytes drives the immune response by spreading to macrophages as they ingest debris. In this situation, the macrophages that are recruited to the lung drive the immune response, rather than resident alveolar macrophages [30].

#### Adaptive immunity

Severe SARS-CoV-2 infection causes a disproportionate decrease in CD4 + T-cells accompanied by alterations of T-cell subsets. Infected patients have increased ratios of naïve to memory CD4 + T-cells and decreased T-regulatory cell populations, especially those modulating allergic responses and autoimmunity [31]. In addition, SARS-CoV-2 infection stimulates Th1 cells, Th2 cells, and Th17 cells, the net effect of which is an elaboration of pro-inflammatory cytokines, such as IL-17,

IL-21, IL-22, and G-CSF. Of these, IL-17 is specifically associated with autoimmunity, whereas G-CSF promotes neutrophilic activation [32]. Patients with severe disease also have increased numbers of LAG3-positive T-cells. This T-cell subset is responsible for IL-10 production, which further reduces the cytotoxic effects of CD8 + T-cells and suppresses IFN-mediated responses [33]. Alterations in T-cells occur in combination with decreased B-cell evolution in germinal centers, as evidenced by increased unmutated IGH clones and plasmablasts [12]. Abnormal circulating monocytes elaborate S100A8 and S100A9, which drive emergency myelopoiesis, suppress normal T-cell function, and delay viral clearance. Increased IFN-α levels also activate NK cells to promote interferon-mediated responses that, when sustained, can lead to NK cell dysfunction and loss [14]. Indeed, emerging data suggest that severe COVID-19 may result from an impaired immune response with delayed viral clearance rather than an excessive immune reaction and post-viral cytokine storm, as has been previously suggested. Timing and location are critical; the lack of upper airway viral clearance allows viral entry to the lower airways and drives dysfunctional emergency responses, essentially combining theories of deficient viral clearance and excessive innate immune response.

### Mechanisms of tissue injury

#### Neutrophil extracellular traps

**Fig. 1:**
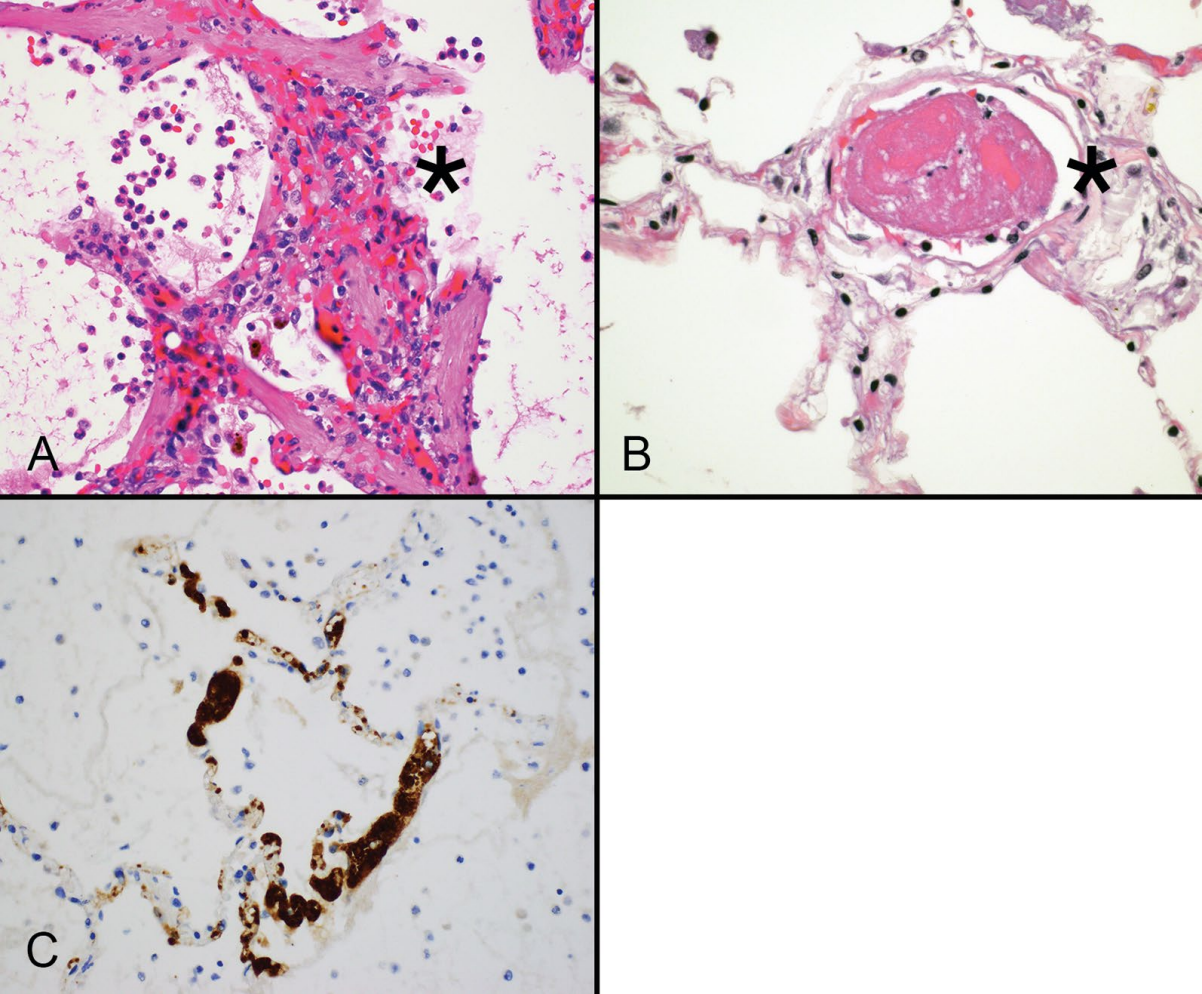
Acute COVID-19 pneumonia features neutrophil extracellular traps (asterisk) that expand the alveolar wall, thereby increasing pulmonary resistance and impairing diffusion. Neutrophil extracellular traps appear as collections of neutrophils, nuclear debris accompanied engorged and damaged capillaries (**A**). Small arterial thrombi (asterisk) are frequently present in the lungs of patients with COVID-19 related pulmonary symptoms (**B**). Capillary beds in the alveolar walls show marked accumulation of platelet-rich thrombi, which is highlighted by immunohistochemistry for CD61. (**A**, **B** hematoxylin and eosin stain)

Neutrophil extracellular traps are three-dimensional lattice-like structures composed of decondensed chromatin strands. Chromatin strands are studded with citrullinated histones, antimicrobial proteins, and cytokines, such that they can entrap and kill pathogens upon release from neutrophils undergoing apoptosis. NETs immobilize and clear organisms by positioning viruses in direct contact with anti-viral proteins. NET formation is associated with increased IL-6, IL-8/CXCL8, RANTES/CCL5 and platelet factor 4, and can be induced directly by viable SARS-CoV-2 [34, 35]. The NET response is one of several critical components of innate immunity, but it can become dysregulated and result in cell injury. Once activated, NETs promote a positive feedback loop that drives platelet aggregation and cytokine release, as well as complement activation [36]. Plasma, tracheal aspirates, lung tissue, and arterial thrombi from patients with severe COVID-19 all contain increased numbers of NETs compared with tissues from patients with pulmonary disease unrelated to SARS-CoV-2 [37, 38]. Importantly, the histologic hallmarks of NETs, namely neutrophils associated with microthrombosis (Fig. 1A), likely account for clinically severe COVID-19 pneumonia [39, 40]. Recognition of the pivotal role NETs play in the pathogenesis of COVID-19 may help identify therapies that inhibit NET induction and become an important strategy for managing critically ill patients [41].

#### Thrombosis

Patients with COVID-19 and ARDS are at risk for systemic endothelial injury and thrombosis involving both venous and arterial systems [42, 43]. They are more likely to develop pulmonary endothelial injury and thrombosis (Fig. 1B) than patients with influenza, and they have higher bleeding rates with elevated markers of systemic injury such as CRP, ESR, fibrinogen and pro-calcitonin [44]. Activation of P-selectin in resting platelets, von Willebrand factor multimers, reduced ADAMTS-13, and increased blood viscosity support the concept that many patients with COVID-19 suffer from abnormal coagulation [45, 46]. The combination of platelet (Fig. 1C) and neutrophil activation also promotes NET formation [47]. Current trends toward implementing anti-coagulation as part of the treatment regimen for severe COVID-19 appear to reduce 30-day mortality without increased bleeding risk [48].

## Clinical features of SARS-CoV-2 infection

The viral incubation period ranges from 4–14 days (median: 5). Many patients are asymptomatic carriers whereas others can become gravely ill. Bennet et al. evaluated 174,568 adults with SARS-CoV-2 infection and reported 20.2% to have severe disease with 18.6% requiring hospitalization during 2020. The overall mortality rate was 11.6%, although death rates decreased from 16.4% in early 2020 to 8.6% by the end of the year [49]. Symptomatic patients typically complain of cough, fever, myalgias, headache, and upper respiratory tract manifestations, whereas approximately 10% have nausea, diarrhea, or a loss of sense of taste and/or smell.

## Risk factors for severe COVID-19

Early in the pandemic, it became clear that advanced age, male gender, specific racial groups (e.g., African American, Latino, Native American), obesity, diabetes mellitus, cardiovascular disease, chronic pulmonary or renal disease, and immunocompromise were associated with more severe disease, especially when multiple risk factors were present [50]. Specifically, patients with severe COVID-19 and high body mass indices tend to have increased IL-6 levels, endothelial injury, IFN activation, and cytokine profiles (e.g., IFN-γ, IL15, IL17, MCP1/CCL2, MIP1-beta/CCL4) that suggest macrophage recruitment [51]. Viral entry may be enhanced among older patients, who tend to have greater expression of TMPRSS2 compared with younger individuals [52].

Several genetic factors may place patients at risk for severe COVID-19. Polymorphisms of OAS1, 2, and 3 represent possible susceptibility loci because they normally activate latent RNAse activity and promote resistance to RNA viruses. Interferon α/β receptor subunit 1 mutations, inborn errors in TLR3 and type I IFN pathway elements, and alterations in TYK2 and IFNAR2 are associated with severe COVID-19 [53–55]. As previously described, TLR7 is crucial sensor of ssRNA that activates human dendritic cells to produce anti-viral IFN [56]. Variants in TLR7 have been reported in up to 2% of males under 60 years of age with severe COVID-19 [57]. Polymorphisms in ACE2 (rs2074192 and rs1978124) have a protective effect while rs2106809 and rs2285666 are associated with increased disease severity [58]. Notably, 10% of patients with critical COVID-19 have autoantibodies that neutralize type I IFN and these antibody levels increase with age, being present in 20% of patients more than 80 years of age who present with severe disease [59].

## Pulmonary disease

### Patterns of inflammation in the lung parenchyma

Much of our understanding of the pathogenesis of severe COVID-19 has been gleaned from autopsy studies, especially during the early months of the pandemic [60]. The combination of real-time tissue banking from blood and autopsy material for use in high-throughput multi-omics techniques led to rapid advancement in the understanding of inflammatory mechanisms underlying disease progression. Imaging mass cytometry showed SARS-COV-2 to predominantly infect lung epithelial cells, although other cell types also contain viral proteins. The levels of ACE2 and TMPRSS2 on AT1 and AT2 cells suggest susceptibility to viral entry, and that increased ACE2-positive cells mirror the development of AT2 hyperplasia in severe disease. AT2 cells and macrophages propagate an IFN-mediated response, neutrophil degranulation, and cellular recruitment via MIP-1α/CCL3 and MIP-1β/CCL4 through the effects of IL-6 and IL-1β. In addition, persistent immaturity of AT2 cells and inadequate surfactant expression may contribute to persistent end-organ failure rather than parenchymal loss and scarring alone.

**Fig. 2:**
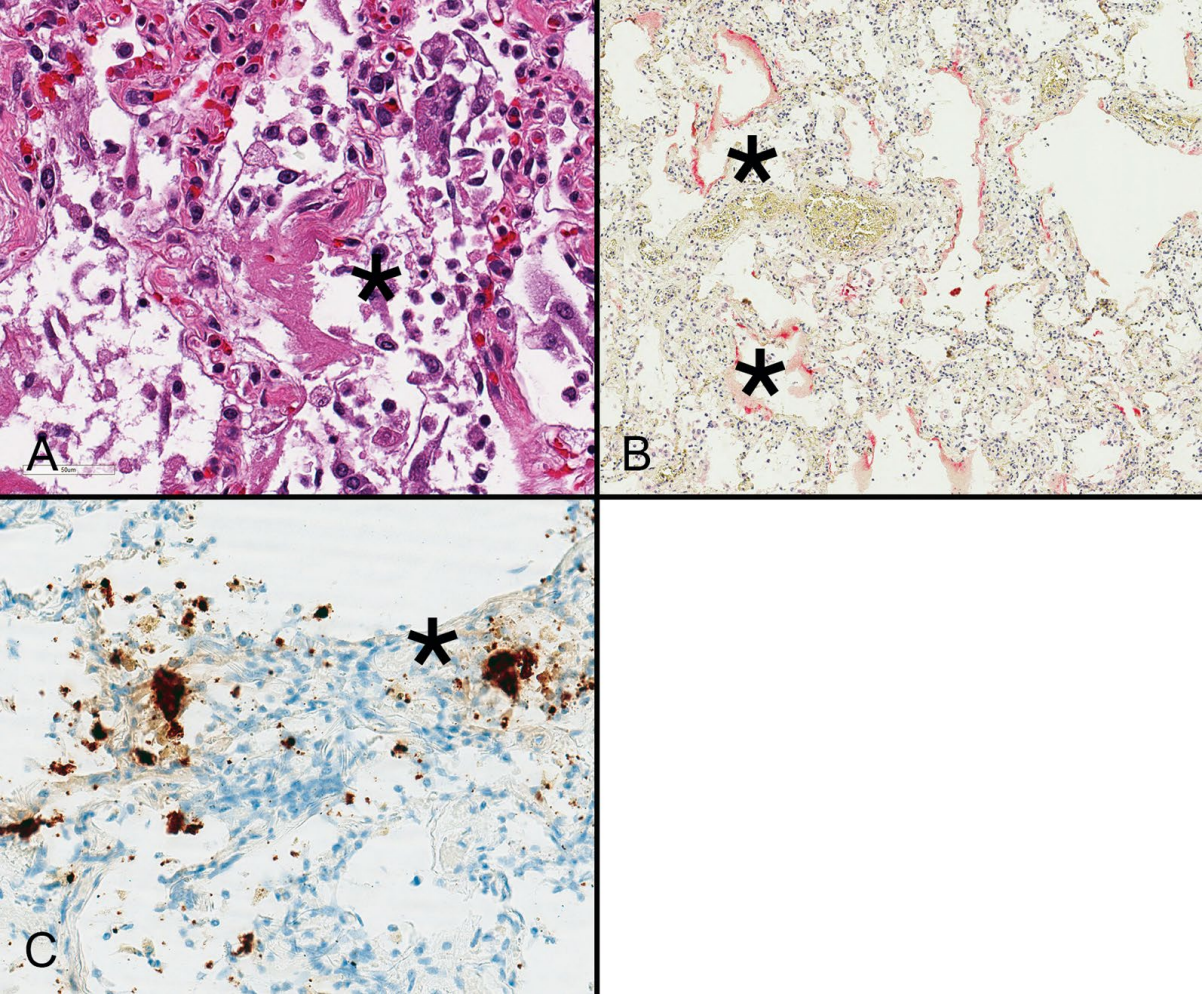
Diffuse alveolar damage is characterized by hyaline membranes (asterisk) resulting from AT1 cell necrosis, as well as exuberant regeneration of AT2 cells during the first week following infection (**A**). Hyaline membranes and epithelial cells show immunoreactivity for spike protein (asterisk) using a red chromogen. **B** These areas also contain viral spike RNA (asterisk) as demonstrated with in situ hybridization using a brown chromogen (**C**). Such cases were also positive by real time polymerase chain reaction (not shown) (**A** hematoxylin and eosin stain)

Viral detection rates in autopsy material tend to be highest in samples obtained from the upper and lower respiratory tracts with less frequent detection in the gastrointestinal tract and failure to identify organisms in the blood, brain, skin, and bile [61]. Most severe and fatal COVID-19 cases involve the lungs with a diffuse alveolar damage (DAD)-type pattern of injury that reflects clinical manifestations of ARDS (Fig. 2A). Some patients develop severe, rapidly progressive DAD resulting from the combined effects of direct viral infection (Fig. 2B, C), immune-mediated injury, and thrombosis, whereas others suffer progressive respiratory failure with organizing DAD. Emerging data suggest that the former situation is associated with higher viral loads, increased cytokine levels, and upregulation of IFN-stimulated genes, whereas the latter is characterized by lower viral loads and less contribution of IFN to the pathogenesis of disease [62].

**Fig. 3:**
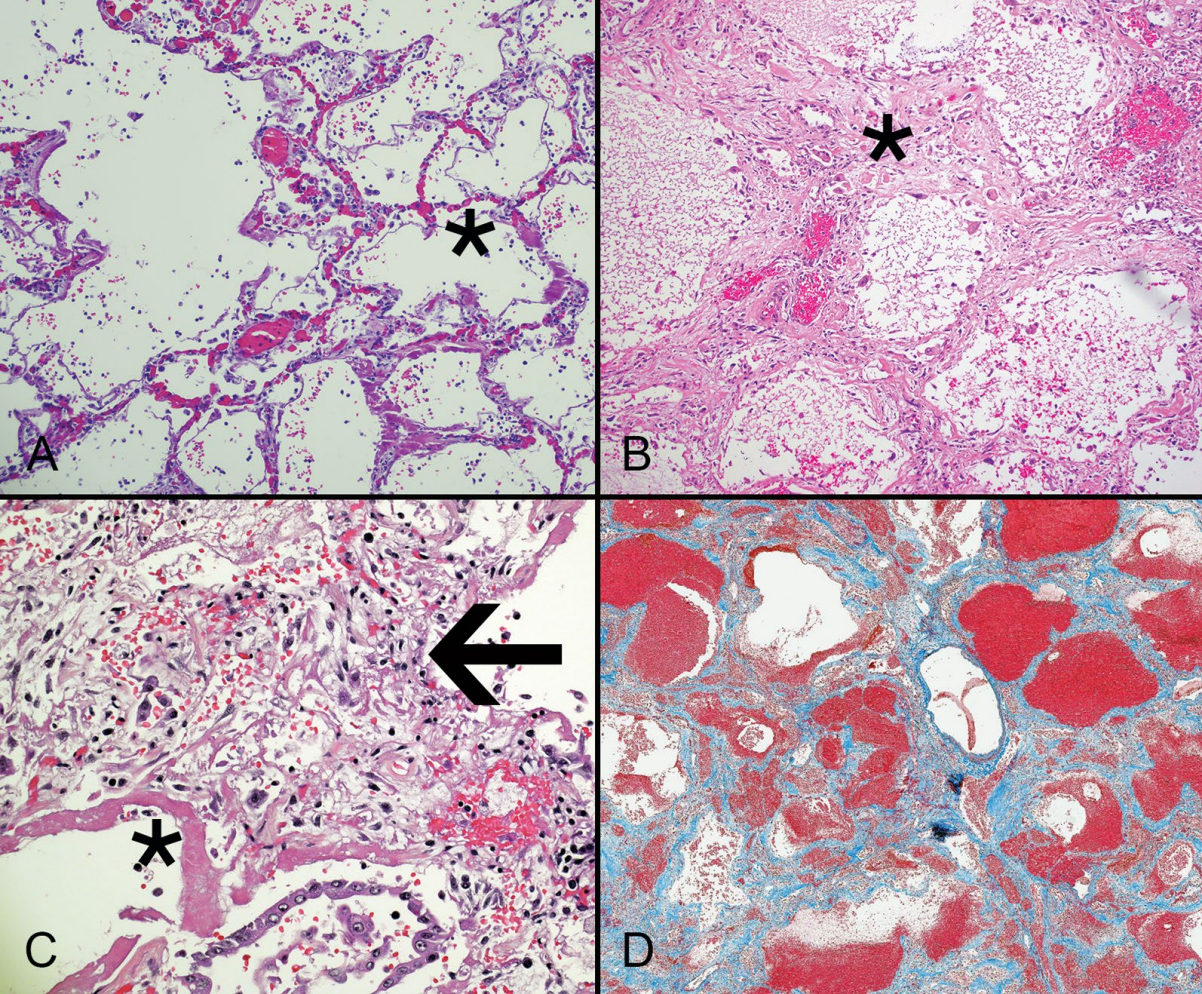
Early COVID-19 causes lung injury characterized by mild congestion of small capillaries in the alveolar walls (asterisk) associated with inflammation and hyaline membranes (**A**). Organizing diffuse alveolar damage in COVID-19 features alveolar walls (asterisk) that are thickened by exuberant fibroblastic proliferations and lined by AT2 cells (**B**). Some cases show juxtaposition of acute injury with hyaline membranes (asterisk), AT2 cell hyperplasia, and interstitial fibroblastic proliferation (arrow) that expands the alveolar walls (**C**). The interstitial fibroblastic proliferation is replaced by dense collagen that stains blue with a trichrome stain (**D**). The alveolar architecture is lost in the late stages of COVID-19 lung disease (**A**–**C** hematoxylin and eosin stain)

COVID-19-related DAD typically features exudative and proliferative phases with hyaline membranes (Fig. 3A) and activated fibroblasts (Fig. 3B), respectively, although many cases show co-mingling of both patterns or a combination of these phases with organizing DAD and fibrosis (Fig. 3C). Some authors have described loss of epithelial cells and an influx of non-resident interstitial macrophages in transitional areas between exudative and organizing DAD [29]. This finding, which has not been reported in ARDS unrelated to COVID-19, suggests SARS-CoV-2 elicits a pro-inflammatory epithelial-monocyte interface [63]. This alteration may predict steroid-responsiveness of pulmonary disease.

Although patients with SARS-CoV-2 infection and ongoing lung injury usually have exudative DAD-type pulmonary disease, some also have changes resembling acute bronchopneumonia that likely reflect bacterial superinfection. AT2 cells associated with hyaline membranes are more likely to contain detectable virus by immunohistochemistry and in situ hybridization. Detection rates are highest within two weeks of infection and decrease over time, although some patients with chronic pulmonary disease still have detectable organisms [64, 65]. Patients who survive the initial phases of severe lung injury can develop pulmonary fibrosis (Fig. 3D) following resolution of ARDS [66, 67].

A subset (< 20%) of COVID-19 patients with pneumonia have “happy hypoxemia” with a paradoxical lack of dyspnea despite poor oxygenation. These cases are characterized by maintained lung compliance, a lack of hypoxic vasoconstriction, and larger intrapulmonary shunts than typically occur in patients with ARDS [68, 69]. Other severely ill patients with airway patency have low respiratory compliance and poorly recruited regions of the lung that require high driving pressures to maintain oxygenation [70–72]. These seemingly distinct clinical subtypes could reflect different timepoints during disease, with the latter occurring when patients develop intrapulmonary thrombosis, as discussed previously [73]. Prone positioning improves airway recruitability in both groups of patients.

### Senescence

COVID-19 can cause organ damage by inducing a macrophage-driven senescent phenotype in epithelial and endothelial cells that results from complement-mediated cytokine induction, thrombosis, and matrix remodeling [74]. Data from human and animal studies have also shown that SARS-CoV-2 stimulates macrophages and T-cells in bronchioloalveolar lavage material, which, in turn, induce pulmonary ciliated and club cells to express markers of senescence such as CDKN2a, CDKN1a, uPAR, CXCL8, IGFBP3 and GDF15 [75].

### Long COVID

A minority of patients, particularly women, continue to experience breathlessness, cough, fatigue, and other symptoms months to years following recovery from COVID-19 [76]. Affected individuals commonly have a measurable diffusion impairment as well as alterations in lung perfusion that may be related to persistent microvascular injury [77]. Pulmonary function tests tend to correlate with the initial disease severity, but do not necessarily bear any relationship to specific symptoms. Patients with long COVID also have altered plasma biomarker levels compared with patients who fully recover. Significant differences in levels of neutrophil markers (e.g., lipocalin-2, matrix metalloproteinases, and repair proteins such as HGF) suggest persistent tissue inflammation and remodeling among patients with long COVID [78].

## Cardiovascular disease

### Endothelial injury

Although endothelial cells express ACE2 and TMPRSS2 and, thus, are potentially infected by SARS-CoV-2, most data suggest that endothelial damage in the context of COVID-19 results from immune activation of endothelial cells by cytokines, kallikrein-kinin activation, IL-6 induction, and complement activation [79]. Any of these factors can promote endothelial cell death via apoptosis or pyroptosis, the latter of which is a mechanism of cell death important to controlling microbial infection. Endothelial injury promotes vascular damage and thrombosis, resulting in dysfunction of multiple organs and organ systems [80, 81].

### Cardiac injury

**Fig. 4:**
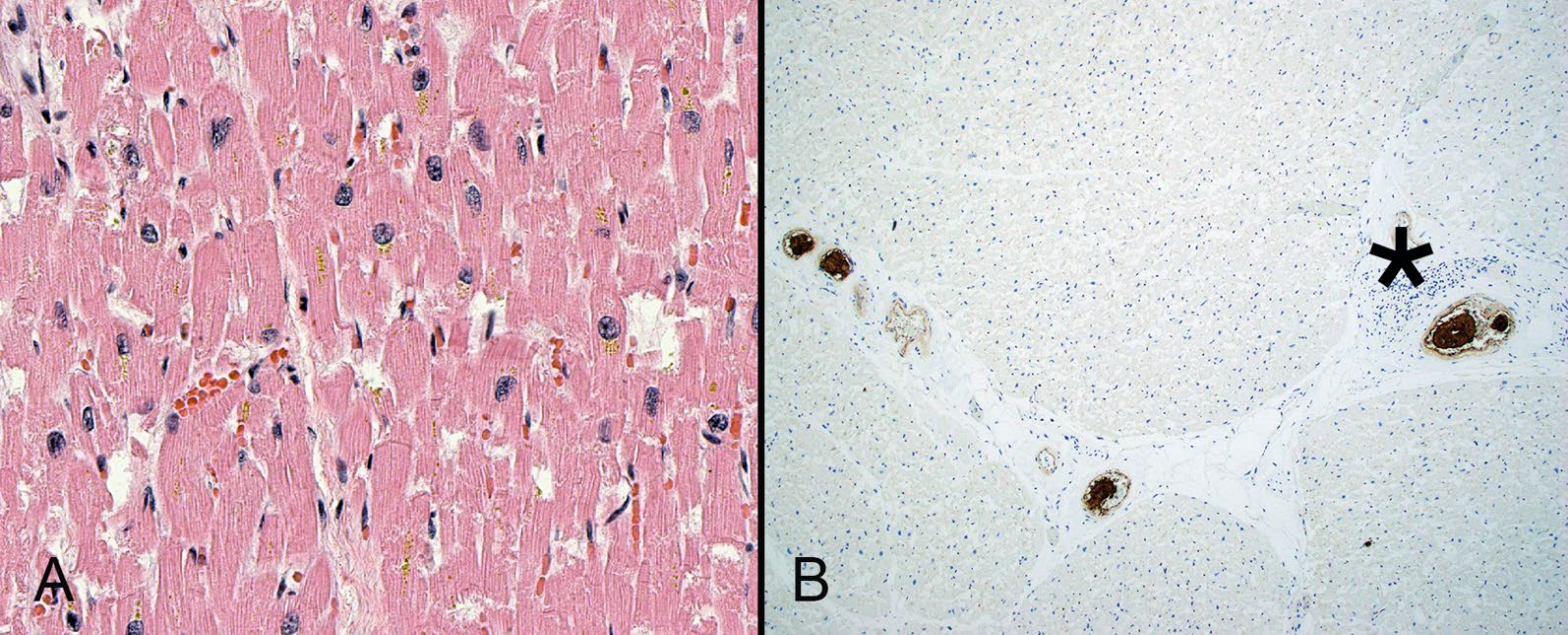
Despite clinical cardiac dysfunction, the histomorphology of cardiac tissue is often relatively unremarkable in patients with severe COVID-19. Sections of the heart from this patient who died of COVID-19 is essentially normal and lacks both inflammation and necrosis (**A**). Microvascular injury, as evidence by intra-myocardial thrombi, may play a role in cardiac disease. Fibrin thrombi (asterisk) are highlighted by immunohistochemistry for CD61 (**B**)(**A**, hematoxylin and eosin)

While early reports included cases of myocarditis, the histopathology of cardiac injury is not frequently associated with inflammation or myocardial necrosis (Fig. 4A). Microvascular injury (Fig. 4B) is central to the proposed mechanism of cardiac injury among patients with SARS-CoV-2 infection [82, 83]. It is likely that the combination of NET formation and platelet activation promote coronary thrombosis to a greater degree than endothelial activation, although different factors may promote injury during various phases of disease [84]. Single cell sequencing studies have not identified the virus in cardiac muscle cells but have shown decreased cardiomyocytes and pericytes relative to endothelial cells [85]. Bulk RNA sequencing studies have demonstrated altered cardiomyocyte differentiation without morphologic abnormalities, raising the possibility that SARS-COV-2 causes organ dysfunction through its effects on differentiation rather than cellular loss [86]. Data from animal models suggest that sinoatrial cells are susceptible to SARS-COV-2 infection and subsequent arrhythmias [87].

## COVID-19 and the brain

Many patients with COVID-19 develop neuropsychiatric symptoms, most of which reflect aberrant inflammatory responses, microvascular injury, and thrombosis in the brain. Single-nucleus transcriptome analyses have failed to detect viral transcripts in the brains of patients with COVID-19 and neurologic symptoms, and western blots fail to detect viral spike protein in these tissues [88]. Rather, transcriptional changes in the cortical microglia suggest increased cellular activation, mobility, and phagocytosis with perturbations in barrier cells of the choroid plexus that allow parenchymal infiltration by peripheral T-cells [89]. These alterations cause persistent neuronal damage affecting synaptic signaling of upper-layer excitatory neurons similar to changes seen in patients with neurodegenerative diseases.

## Gastrointestinal effects of SARS-CoV-2

### The small and large intestines

Both ACE2 and TMPRSS2 are highly expressed in the gastrointestinal tract and, thus, it is not surprising that gastrointestinal infection by SARS-CoV-2 is relatively common [4, 90, 91]. In fact, more than one-third of patients with pulmonary symptoms have detectable virus in their stool samples, which may persist for weeks to months following onset of COVID-19-related symptoms [92]. Approximately 20% of infected patients develop gastrointestinal symptoms, particularly abdominal pain, bloody diarrhea, or non-bloody diarrhea. The severity of gastrointestinal manifestations does not necessarily correlate with the severity of pulmonary symptoms [93].

**Fig. 5:**
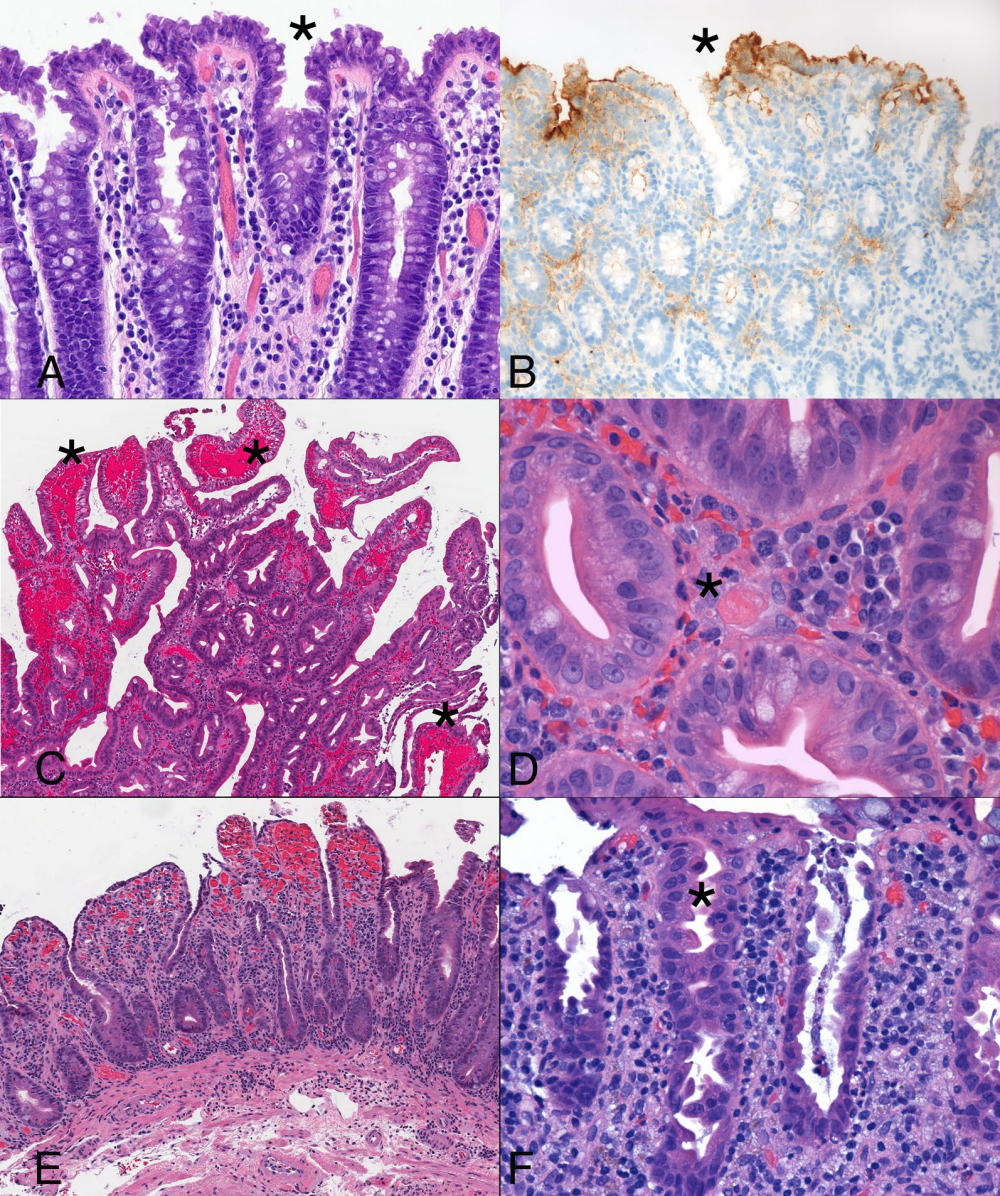
A colonic resection specimen from a patient with COVID-19 and no gastrointestinal symptoms shows subtle features of viral infection. The colonic crypts are slightly dilated near the surface and have a slightly tufted appearance (asterisk) that reflects clustered epithelial cells with nuclear crowding (**A**). The same areas show strong signal for spike protein RNA by in situ hybridization (asterisk) using a brown chromagen (**B**). Another patient with mild respiratory symptoms due to COVID-19 developed gastrointestinal bleeding. Biopsy samples from the duodenal mucosa shows normal villous architecture with patchy hemorrhages (asterisk) and regenerative epithelium with cytoplasmic depletion. **C** Platelet-rich thrombi (asterisk) are present in vessels (**D**). A third patient with COVID-19 developed severe diarrheal symptoms presumably due to the pro-inflammatory state promoted by the virus. Samples obtained from the jejunum display mild villous shortening with striking crypt hyperplasia and regenerative epithelial changes unaccompanied by substantially increased inflammation (**E**). Colonic samples from the same patient show a similar degree of epithelial cell injury without increased inflammation. Dilated crypts (asterisk) contain mucin-depleted cells with eosinophilic cytoplasm and disorganized nuclei (**F**)

In vitro models utilizing intestinal organoids have shown the virus to directly infect colonic epithelial cells, then spread rapidly via cell-to-cell transmission [94]. Colonic infection by SARS-CoV-2 elicits cytologic changes unaccompanied by substantial epithelial cell necrosis or mucosal inflammation [95]. Infected cells tend to be crowded with a tufted appearance, mucin-depletion, and blebs of eosinophilic cytoplasm, particularly in the superficial crypts and surface epithelium (Fig. 5A). Cells that display these cytologic abnormalities show strong, diffuse signal for the SARS-CoV-2 spike protein by in situ hybridization, immunopositivity for the spike protein, and ultrastructural evidence of viral infection (Fig. 5B) [95].

It is worth noting that direct viral infection of the gastrointestinal tract is likely a transient event that causes few, or no symptoms. Most gastrointestinal manifestations of COVID-19 are probably secondary to cytokine elaboration, hypercoagulability, and microvascular injury triggered by the virus, rather than direct viral cytotoxicity. Westerhoff, et al*.* evaluated gastrointestinal specimens from 25 patients with detectable SARS-CoV-2 infection by nasopharyngeal testing but could not consistently demonstrate detectable virus in the gastrointestinal tract, even when inflammatory or ischemic-type changes were present in the samples [96]. Zhang et al*.* also failed to identify SARS-CoV-2 by either immunohistochemistry for the nucleocapsid protein or in situ hybridization for viral spike protein RNA when evaluating 28 bowel resection cases from patients with COVID-19 [97].

Patients with SARS-CoV-2 infection are prone to coagulopathies and, thus, gastrointestinal manifestations of COVID-19 most commonly include ischemic-type changes with thrombi in the small and/or large intestines [98–103]. These changes may develop days to weeks after viral detection and onset of pulmonary symptoms and, thus, ancillary studies generally fail to demonstrate the organism in biopsy and resection specimens [104, 105]. Patchy mucosal hemorrhage and fibrin thrombi in small capillaries may be present in biopsies obtained upon onset of gastrointestinal symptoms, at which point the background mucosa may be normal or show epithelial cell regeneration with cytoplasmic depletion and mild nuclear enlargement (Fig. 5C, D). Resection specimens show more extensive ulcers with thrombi in small and large submucosal veins, as well as patchy necrosis of the muscularis propria. In the previously cited study, Zhang et al*.* reported frequent fibrin thrombi, strands of fibrin in non-occluded enteric vessels, and a high rate (60%) of pneumatosis in patients with ischemic enterocolitis due to COVID-19 compared with patients who had ischemic enterocolitis due to other causes. The same authors also described a minority of cases that showed patchy fibrosis of the outer muscularis propria, suggestive of chronic ischemic injury [97].

Infection with SARS-CoV-2 can promote epithelial cell damage in the gastrointestinal tract, resulting in diarrheal symptoms rather than signs and symptoms of bleeding (Fig. 5E, F). The virus downregulates ACE2 expression on the gut epithelium, which diminishes the counterregulatory effects of ACE2 on angiotensin II-mediated signaling and increases inflammation [106]. Decreased ACE2 expression disrupts ACE2-mediated tryptophan absorption and negatively affects amino acid homeostasis. In addition, lower levels of ACE2 lead to diminished mTOR-mediated expression of antimicrobial proteins at the luminal surface [106, 107]. The virus also promotes a pro-inflammatory state through ACE2-independent mechanisms that involve elaboration of pro-inflammatory cytokines [82].

### The pancreas

Type I diabetes mellitus is characterized by immune-mediated destruction of the islets of Langerhans in genetically susceptible patients, most of whom have either HLA-DR3 or HLA-DR4 haplotypes [108]. Its pathogenesis is not entirely clear, but there is ample evidence that some RNA viruses, namely rotaviruses and coxsackieviruses, contribute to disease development and persist in islet β-cells for prolonged periods of time. Viral entry into β-cells can cause direct cytotoxic injury, resulting in cell death. Alternatively, elaboration of viral antigens combined with β-cell-specific antigens, evokes an immune response that results in β-cell destruction, especially in the context of increased IFN production and upregulation of class I major histocompatibility complexes [109].

Early in the pandemic, it became clear that patients with diabetes mellitus were at risk for more severe SARS-CoV-2 infections [50]. However, emerging data also suggest an increased risk for new-onset diabetes mellitus, as well as metabolic complications of pre-existing diabetes mellitus among patients with resolving COVID-19. The effects of COVID-19 on glucose homeostasis are likely multifactorial. β-cells express ACE2 and TMPRSS2, as well as other entry factors, such as neuropilin-1 (NP-1), transferrin receptor (TFRC), FURIN, CTSL, and dipeptidyl peptidase-4 (DPP-4) [110, 111]. Like rotaviruses and coxsackieviruses, SARS-CoV-2 can directly damage β-cells and induce apoptosis once inside the cell [110, 111]. However, it also downregulates insulin gene transcription, while glucagon and other peptides are upregulated, and causes transdifferentiation of β-cells to express both endocrine and acinar markers [112]. Viral binding depletes ACE2 at the cell surface and diminishes its counterregulatory effects on angiotensin II-mediated signaling. Activation of the renin-angiotensin pathway further promotes insulin resistance, inflammation, and β-cell dysfunction [113].

## Dermatologic manifestations of COVID-19

The cutaneous manifestations of COVID-19 are broadly grouped in six patterns of injury: confluent erythematous/maculopapular/morbilliform rash, urticarial rash, papulovesicular exanthem, Chilblain-like acral pattern, livedo reticularis/racemose-like pattern, and purpuric pattern [114]. These patterns of injury likely reflect cutaneous reactions to circulating viral antigens and thus, typically feature endothelial injury and perivascular inflammation of dermal vessels. Nearly 50% of patients with rashes related to SARS-CoV-2 have erythematous or maculopapular eruptions that develop after the onset of systemic symptoms. These lesions tend to be pruritic and show a symmetrical distribution over the trunk with progression in a centrifugal fashion [115]. Although data are scarce, these eruptions seem to reflect some type of vascular injury, often with perivascular lymphocytic or neutrophilic infiltrates [116, 117]. Urticarial rashes are most pronounced over the trunk and limbs and may be accompanied by angioedema or urticarial vasculitis [118]. They usually present at the same time as other systemic symptoms although they can precede pulmonary disease manifestations in occasional patients. Papulovesicular exanthems typically feature scattered vesicles over the trunk with, or without, pruritis [114]. Biopsies reveal epidermal acantholysis with ballooned keratinocytes and endothelial inflammation in dermal vessels [119].

Chilblain-like acral pattern, livedo reticularis/racemose-like pattern, and purpuric pattern eruptions typically feature both endothelial damage and thromboses, possibly due to IFN-mediated inflammatory reactions or endothelial injury secondary to circulating viral particles [120]. Chilblain-like lesions appear as violaceous plaques predominantly located on the feet with occasional involvement of the hands or ears. These lesions are commonly painful or itchy and, unlike other dermatologic manifestations of COVID-19, tend to occur in patients with minimal, or no, systemic symptoms [121]. The livedo reticularis/racemose-like pattern appears as lace-like dusky patches of blue discoloration and likely reflects slow blood flow secondary to thrombosis of small superficial vessels in the dermis. Magro et al. described three patients with this pattern of injury, all of whom had a pauci-inflammatory microthrombotic vasculopathy, leading the authors to hypothesize that thrombosis resulted from complement activation in the absence of an appropriate IFN-related immune response [120, 122]. In contrast, the purpuric pattern of injury represents a true vasculitis likely secondary to direct endothelial damage by viral particles [123]. This hemorrhagic blistering or nodular rash tends to occur in intertriginous regions or show an acral distribution. Biopsy samples demonstrate a leukocytoclastic vasculitis with fibrin thrombi and injured endothelial cells [124].

## Conclusions

The normal anti-viral response initially involves type I IFN elaboration from NK cells and CD8 + T-cells, ultimately killing infected cells and clearing the virus, whereas severe viral infections elicit IL-8/CXCL8 activation that promotes neutrophil chemotaxis, activation, and NET formation. This process is regulated by plasmacytoid dendritic cells that both produce type I IFN and decrease IL-8/CXCL8 production [125]. Dysregulation of these immune responses is central to development of COVID-19, as the combined effects of lymphopenia, reduced NK cells and CD8 + T-cells, and decreased plasmacytoid dendritic cells diminish type I IFN-mediated responses and causes a shift toward IL-8/CXCL8-induced neutrophil recruitment [126]. Continued induction of MCP1/CCL2, CXCL10, IL-6, TNF-α and IL-1β combined with terminal complement activation further stimulate neutrophil activity, culminating in NET formation and an environment conducive to inflammatory thromboses [126]. These perturbations have similar manifestations in multiple organ systems, which generally show pathologic findings related to microvascular injury and thrombosis of large and small vessels. However, the pulmonary findings in patients with severe COVID-19 are generally more pronounced than those of other organs. Not only do they feature inflammatory thromboses and endothelial injury, but much of the parenchymal damage stems from failed maturation of alveolar pneumocytes, interactions between AT2 cells and non-resident macrophages, and a greater degree of NET formation. Understanding these mechanisms of injury is important to development of future therapies for patients with COVID-19, many of which will likely target specific components of the immune system, particularly NET induction, pro-inflammatory cytokines, and specific subpopulations of immune cells.

## Data Availability

Not applicable.
